# Steroid‐induced inflammatory papillary hyperplasia?—A new entity in a non‐denture wearing patient

**DOI:** 10.1002/ccr3.7487

**Published:** 2023-06-07

**Authors:** Abhishek Gupta, Ram Sudan Lamichhane, Anju Redhu

**Affiliations:** ^1^ Department of Oral Medicine and Radiology, School of Dental Sciences Chitwan Medical College Bharatpur Nepal; ^2^ Department of Oral Pathology KIST Medical College and Teaching Hospital Lalitpur Nepal; ^3^ Department of Oral Medicine and Radiology PGIDS Rohtak India

**Keywords:** complete denture, hard palate, inflammatory papillary hyperplasia, oral mucosal lesion, steroids

## Abstract

**Key Clinical Message:**

Inflammatory papillary hyperplasia can be seen in a non‐denture‐wearing patient also therfore other etiologies need to be explored as well.

**Abstract:**

Inflammatory papillary hyperplasia (IPH) is a benign lesion of the palatal mucosa, usually found in denture wearers. This case history report describes an example of the dentate patients with no history of wearing maxillary prostheses and highlights the importance of professional awareness to diagnose IPH among non‐denture‐wearing patients.

## 1 INTRODUCTION

Inflammatory papillary hyperplasia (IPH) is an oral mucosal lesion (OML).^1^ It is a painless, firm, pink or red, nodular mucosal growth. As IPH is mostly asymptomatic, it is diagnosed incidentally.^2^, ^3^ Hard palate is the only site involved by IPH in almost all cases^1^; however, it may occasionally extend to the mucosa of the residual ridges.^2^ IPH is more prevalent in males and usually in around 79% of the cases located in anterior region of maxilla.^4^ In general, IPH is caused by the irritation of removable or complete partial dentures of maxillary arches. To the best of our knowledge, in only three of the reported cases it has also been found in dentulous patients with no history of any denture.^1^, ^5^, ^6^ IPH has also been associated with predisposing factors like, smoking, tobacco, alcohol and candida infection. Various systemic diseases like diabetes mellitus are considered to be responsible for IPH.^7^ In this case report, IPH is described in a patient who has never worn a maxillary denture, and the causes of this unusual presentation are identified.

## 2 CASE HISTORY/EXAMINATION

**FIGURE 1:**
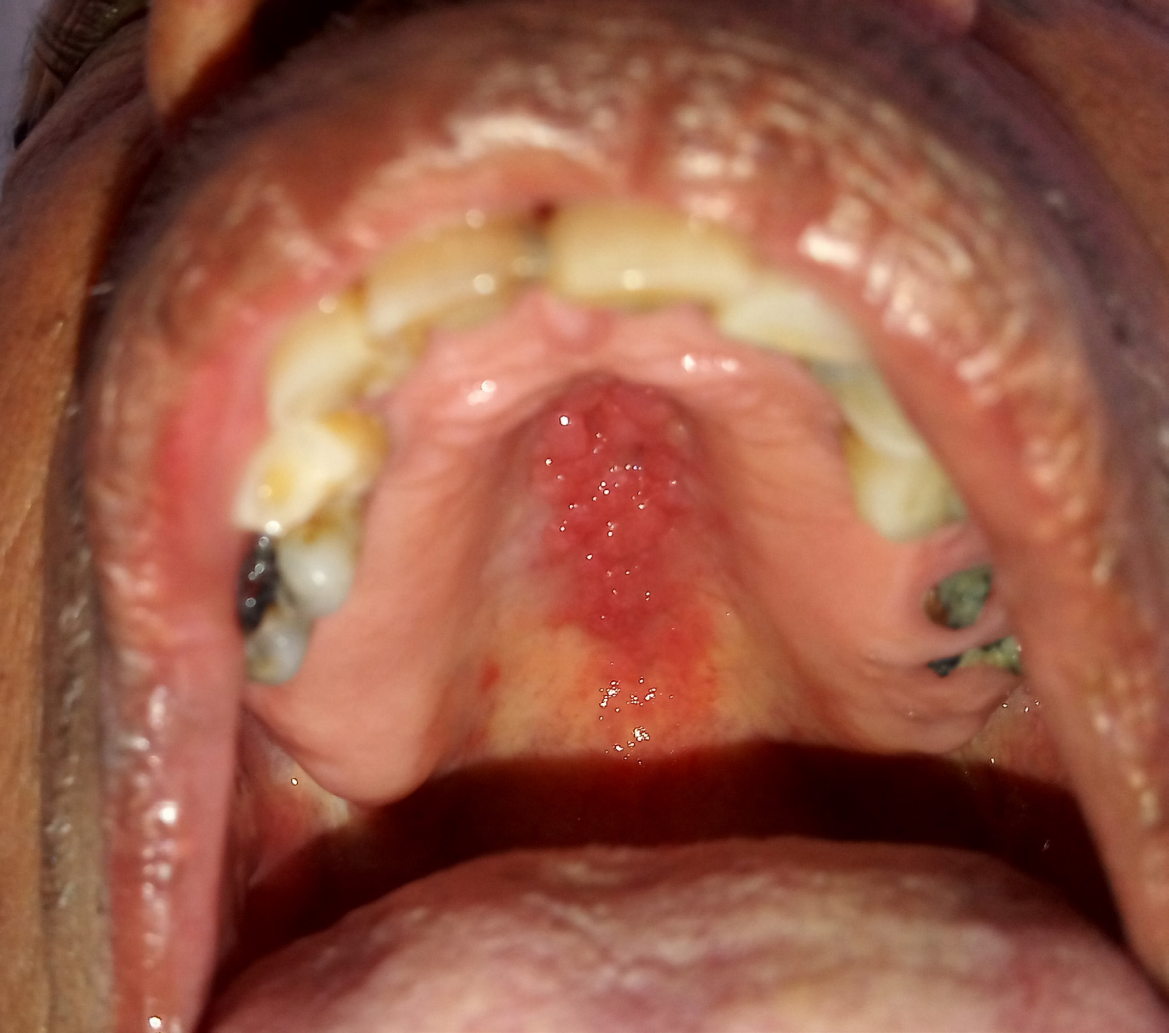
Showing the multiple papillary projection with respect to mid palatal region.

A 75‐year‐old man reported to the department of oral medicine and radiology with the chief complaint of growth in the mid‐palatal region (Figure 1) since 4 months. The patient had a significant history of bronchial asthma since 10 years and was on inhalational steroidal therapy. There was no other relevant medical history reported by the patient. Also, the patient had no history of ever wearing a removable prosthesis. Clinical examination revealed a dentulous maxilla with a mid‐palatal lesion characterized by multiple papillary projections covering approximately about 1 cm × 1 cm area of palate. The overlying mucosa appeared erythematous. There was neither a history of bleeding or pus discharge nor was it present on palpation. The provisional diagnosis of IPH was arrived on the basis of the clinical appearance and examination. The patient was prescribed a topical application of antifungal gel, (clotrimazole 1% w/v) QID for 2 weeks, following which, the patient had reported (telephonic communication) a decrease in size of the lesion. The patient had not visited our department amid COVID‐19 situation.

## 3 DISCUSSION

Inflammatory papillary hyperplasia is a clinical entity or a lesion that is most commonly associated with the patients of maxillary complete denture. Previous literature reviewed has reported most of the cases with the ill‐fitting denture or long term usage /night wearers as the possible etiological factor. However, IPH can occur in non‐denture wearers too. IPH occurring in a non‐denture wearer is a rare entity, as till now only 3 cases have been reported. The first case was in the year 1990, in which the patient presented was a 10‐year‐old black girl without a history of a dental prosthesis.^5^ It is conjectured that poor oral hygiene and a habit of mouth breathing contributed to the occurrence of IPH in this patient. The lesion was surgically removed, and the patient was followed up for a period of 18 months without recurrence of the lesion. The second case was in a 60‐year‐old man with the hard palatal nodular lesion with the history of tobacco smoking, poor oral hygiene and having no history of ever wearing a removable prosthesis. The diagnosis of IPH was made with a palatal mucosal biopsy, and the patient was advised to quit smoking. The topical application of miconazole 2%, three times a day for 3 weeks, was prescribed.^6^ The third case was a 29 years old healthy woman, where on a routine intraoral examination, a red pebbly plaque with irregular and poorly defined borders on palatal vault was found. Nystatin suspension was prescribed. A re‐examination was done after 1 week, and reduction in lesion size and erythema was observed.^8^ The case which has been reported here was that of a 75 year old man with no history of denture who had developed lesion in mid‐palatal region. The patient had the history of usage of inhalational steroid; hence, it can be hypothesized that this would have led to the development of the lesion. Steroids have been associated with the candida infection and chronic hyperplastic candidiasis of oral mucosa. Therefore, it may be also associated with the development of IPH. There have been several studies conducted in the past that have suggested for the involvement of various bacterial and fungal infections for inflammation and irritation of the oral mucosa and gastrointestinal mucosa.^9^, ^10^ *Helicobacter pylori* have been liked with the development of various oral mucosal lesions including lingual papillary hyperplasia.^11^ Similary, *Helicobacter pylori* (*H. pylori*) could also be associated with the development of IPH. In multiple studies by M Cześnikiewicz‐Guzik et. al,^10^ they have concluded that there is no significant correlation between the occurrence of *H. pylori* in stomach.^9^

Other factors such as vitamin deficiency and causes for immunosuppression needs to be further explored as they might have a link with IPH. Yet, further research is required in this regard about the etiopathogenesis of IPH.

## 4 CONCLUSION

To conclude, this case emphasizes the awareness among oral diagnosticians about the IPH in non‐denture patients and other possible etiological factors.

## AUTHOR CONTRIBUTIONS

**Abhishek Gupta:** Conceptualization; data curation; formal analysis; investigation; methodology; project administration; resources; supervision; validation; visualization; writing – original draft; writing – review and editing. **Ram Sudhan Lamichhane:** Conceptualization; data curation; formal analysis; methodology; visualization; writing – review and editing. **Anju Redhu:** Methodology; supervision; validation; visualization; writing – original draft; writing – review and editing.

## FUNDING INFORMATION

None.

## CONFLICT OF INTEREST STATEMENT

The authors declare that there is no conflict of interests regarding the publication of this paper.

## CONSENT

Written informed consent was obtained from the patient to publish this report in accordance with the journal's patient consent policy.

## Data Availability

The data is available with the correspondence author and can be availed on request.
